# Achieving asymmetry and trapping in diffusion with spatiotemporal metamaterials

**DOI:** 10.1038/s41467-020-17550-5

**Published:** 2020-07-24

**Authors:** Miguel Camacho, Brian Edwards, Nader Engheta

**Affiliations:** Department of Electrical and Systems Engineering, University of Pennsylvania, Philadelphia, PA 19104 USA

**Keywords:** Electrical and electronic engineering, Metamaterials, Applied physics

## Abstract

The process of diffusion is central to the ever increasing entropic state of the universe and is fundamental in many branches of science and engineering. Although non-reciprocal metamaterials are well developed for wave systems, the studies of diffusive metamaterials have been limited by their characteristic spatial inversion symmetry and time inversion antisymmetry. Here, we achieve large spatial asymmetric diffusion characteristics inside a metamaterial whose material parameters are space- and time-modulated. Inside such a spatiotemporal metamaterial, diffusion occurs as if the material had an intrinsic flow velocity, whose direction is dictated by the relative phase between the modulations of the conductivity and capacity. This creates dramatic out-of-equilibrium concentrations and depletions, which we demonstrate experimentally for the diffusion of electric charges in a one-dimensional electrical system composed of an array of space-time-modulated variable capacitors and switches. These results may offer exciting possibilities in various fields, including electronics, thermal management, chemical mixing, etc.

## Abstract (web summary)

Being able to manipulate the temporal evolution and spatial distribution of diffusive quantities would provide exciting possibilities for applications. Here, the authors show that one can achieve large spatial asymmetric diffusion characteristics inside a metamaterial whose material parameters are space- and time-modulated.

## Introduction

Diffusion is a physical mechanism that dictates how a wide range of physical systems evolve in time. From the mixing of chemical solutions to the transfer of heat and the distribution of charge on a surface, all physical phenomena based on diffusion tend to an equilibrium. This steady state is ultimately dictated by the increase of entropy of the system with time. It has been established in the past that some special materials have the ability to diffuse differently along different axes, a phenomenon known as anisotropic diffusion^1^. However, it can be shown that diffusion phenomena in homogeneous materials obey a parity symmetry, which means that diffusion, in homogeneous media, occurs identically along the positive and negative directions of a given axis^2^. One way to break such a symmetry has been shown to be the use of a moving medium; for instance, showing an asymmetric diffusion of chemical solutions within airflow^3^. Therefore, let us pose the following question: can we design an effectively homogenized material in which diffusion would have a spatially directional preference?

In the context of wave propagation, researchers have achieved platforms that support the propagation of waves along a preferred direction by breaking the time-reversal symmetry of the wave equation. One way to do this is by introducing external mechanisms to bias the wave-supporting media such as medium velocity for the case of acoustic waves^4^ or external magnetic fields for electromagnetic waves^5–10^. Also, the use of nonlinear materials provides a route towards nonreciprocity^11,12^. More recently, researchers in the field of electromagnetics have extensively explored the use of metamaterials whose effective constitutive parameters (permittivity and/or permeability) vary in space and time to allow non-reciprocal behavior^4,13–17^. These non-reciprocal wave systems can be used to achieve persistent near-field electromagnetic flow of thermal energy through coherent radiation^18^. However, in contrast to the laws that govern propagation of waves, the diffusion equation is inherently not symmetric with respect to a time reversal.

For the case of diffusion, the concept of metamaterials^19^ led researchers to explore the flow of heat through microscopically patterned structures, achieving effective infinite thermal conductivities with applications for cloaking^20^, negative effective thermal conductivities^21^, and heat flux guiding^22^. Exploiting a parallelism with transformation optics^23–25^, it was shown that, in general, heat can be manipulated analogously to light by microscopically designing the conductivity tensor of a metamaterial^25^. Recently, researchers have achieved motionless temperature profiles between two counter-moving fluids by exploiting the anti-time symmetry of the diffusion equation^26^.

In this study, we introduce a paradigm to control those physical quantities that obey the diffusion equation. This is done using a metamaterial comprised of spatiotemporally modulated structures capable of breaking spatial symmetry. We apply homogenization techniques in such media to obtain effective parameters of conductivity and capacity. Such effectively homogenized material exhibits unprecedented control over diffusion features. Our proof-of-concept experiments demonstrate and verify our ideas. The concepts presented here may be applicable to any physical system governed by the diffusion equation.

## Results

### Diffusion in conventional materials

In the diffusion equation, the material capacity relates the potential (temperature, voltage, etc.) required to store a certain amount of the conserved quantity of interest (thermal energy, charge, etc.) per unit volume and a conductivity term, which relates the transport of that quantity between two points of different potential. For the case of heat, the pertinent material quantities are heat capacity and thermal conductivity, whereas for the case of electric charge diffusion they are electrical capacitance and electrical conductivity.

**Fig. 1:**
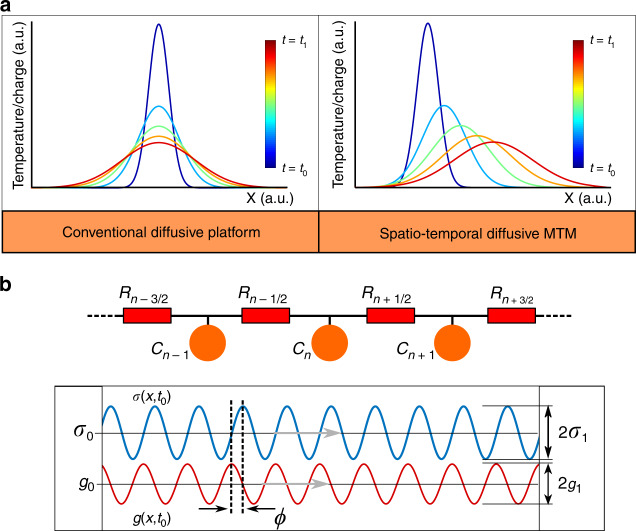
Conceptual representation of controlling diffusion with metamaterial (MTM) platform. **a** Comparison between the time evolution of the typical diffusion process in a conventional material and in our proposed spatiotemporal metamaterial, the latter showing an effective spatial asymmetry in diffusive flow. **b** Generalized schematic representation of the metamaterial platform for periodic spatiotemporal modulation of the two governing macroscopic material properties: conductivity and capacity with a relative phase difference $\phi$ that controls the direction of the effective flow, as described in the text.

Diffusion can be macroscopically understood in terms of Fick’s diffusion equation^27,28^, which in its most general form for the one-dimensional (1D) scenario can be written as

1 $$\frac{{\partial q\left( {x,t} \right)}}{{\partial t}} = \frac{\partial }{{\partial x}}\left( {\sigma \frac{\partial }{{\partial x}}\left( {g\,q\left( {x,t} \right)} \right)} \right)$$

where $\sigma$ and $g$ correspond to the conductivity and the inverse of capacity of the medium, respectively, and both can in general be functions of space and/or time. In its simplest form when these two parameters are assumed to be independent of time and space, the above equation is simplified to its well-known form

2 $$\frac{{\partial q\left( {x,t} \right)}}{{\partial t}} = D\frac{{\partial ^2}}{{\partial x^2}}q\left( {x,t} \right)$$

where $D = \sigma g$ is the diffusion coefficient. The solution of such diffusion equation is well known and is depicted in the left panel of Fig. 1a, which is associated with conventional homogeneous diffusive media. Conventionally, to engineer the diffusive response of the system, one would judiciously choose the right spatial distribution of conductivity and capacity by selecting a combination of conductors, insulators, and structures with proper capacity.

### Diffusion in spatiotemporal metamaterials

We ask the following question: can we propose a medium over which the diffusion occurs in a “biased” asymmetric manner, thus breaking the diffusion spatial symmetry, as sketched in the right panel of Fig. 1a? As we show below, this is possible using a properly designed spatiotemporally varying diffusive metamaterial, in which both the temporal and spatial variation of conductivity and capacitance parameters are judiciously performed, enabling such control over diffusion. This allows modification of the temporal and spatial response of the diffusion phenomena, leading to the breakage of spatial symmetry and even allowing for concentration and accumulation of the quantity of interest with time (such as charge or temperature). Moreover, this platform also provides control over the transient as well as the steady-state behavior of the solutions to the diffusion equation.

For that purpose, let us now consider a metamaterial composed of an infinite chain of miniaturized parallel capacitive elements connected by series finite-conductivity elements as depicted in Fig. 1b. We assume both conductive and capacitive elements to be functions of time (*t*) and space (*x* as, e.g., $\sigma \left( {x,t} \right) = \sigma _0 + \sigma _m\sin \left( {kx - \omega t} \right)$ and $g\left( {x,t} \right) = g_0 + g_m{\mathrm{sin}}\left( {kx - \omega t + \phi } \right)$, both with the same modulation wavelength and frequency, where $0 {\,} < {\,} \sigma _m {\,} < {\,} \sigma _0$ and $0 {\,} < {\,} g_m {\,} < {\,} g_0$. Inserting these functions into Eq. (1), we homogenize the equation in space and time, when the period of temporal variation of the material parameters is much smaller than the response time of the material, dictated by the average value of those modulated parameters (see Supplementary Note 1), obtaining the effective diffusion equation^29^

3 $$\frac{{\partial \tilde q\left( {x,t} \right)}}{{\partial t}} = \tilde D\frac{{\partial ^2}}{{\partial x^2}}\tilde q\left( {x,t} \right) - v\frac{{\partial \tilde q\left( {x,t} \right)}}{{\partial x}}$$

where $\tilde q\left( {x,t} \right)$ is the time-averaged charge density (averaging is done over the period of modulation, which is a much shorter time than the diffusion time), and $\tilde D = \sigma _0g_0 + \frac{1}{2}\sigma _mg_m\cos \left( \phi \right)$ and $v = \frac{1}{2}k\sigma _mg_m\sin \left( \phi \right)$ are the homogenized coefficients obtained by assuming very rapid oscillations of the constitutive parameter modulations (and smooth spatial variations of $q\left( {x,t} \right)$). This partial differential equation is known as the advection–diffusion (or Smoluchowski) equation^30^, which corresponds to the diffusion in a medium that moves along the *x* direction with a velocity $v$ with respect to the observer. This velocity term breaks the spatial symmetry of the system (*x* and −*x* are no longer interchangeable). The parameters $v$, which represents the direction of the “medium bias,” and $\tilde D$ can be controlled with the relative phase $\phi$. We note that this temporal homogenization is valid when the modulation’s temporal period is much smaller than the temporal rate of change of the diffusion mechanism, dictated by $\tilde D$, and the spatial distribution of the diffusive quantity (specifically its second derivative). This fact makes it challenging to obtain a universal rule for the range of validity of the homogenization, although we provide a number of validating examples in Supplementary Figs. 1 and 2 for several initial conditions.

This homogenization results in two arguably unexpected outcomes. First, the effective velocity obtained in Eq. (3) depends on the relative phase of the modulation of conductivity and capacity terms, and depends neither on the frequency nor direction of the underlying spatiotemporal modulations (see Supplementary Movies 1 and 2). Second, although the metamaterial’s response is equivalent to that of a moving flow, there is no actual mechanical or physical flow. The effective velocity can in fact be discontinuous, as we will explore later. Extending this idea, one can also design a metamaterial platform made of multiple domains with different effective velocity distributions (even opposite directions). This idea can lead to a carefully designed flow structure with a tailored steady-state solution.

In the following, let us introduce two different mechanisms that take advantage of this effective velocity to produce large concentrations of the diffusive quantity. First, let us imagine a finite size metamaterial, which is insulated against the flow at both of its boundaries at which the electric current (or the heat flux) must therefore vanish. In the absence of biasing effective velocity, the charge density (or thermal energy) will eventually come to equilibrium with a uniform distribution. However, if the biasing effective velocity is non-zero, charge (or thermal energy) will be driven towards one end of the structure, accumulating until a balance is reached between the two forces acting on them: the diffusive force, which is trying to reduce the accumulation, and the force due to the biasing effective velocity, which pushes it towards the insulating interface. The stationary solution in this case takes the form of an exponential $q\left( x \right) = q_0\exp \left( {\alpha \,x} \right) + q_1$, where $\alpha = v/\tilde D$, similar to the stationary solutions found when drift and diffusion currents balance in solid-state physics^31^.

### Experimental validation of asymmetric diffusion

**Fig. 2:**
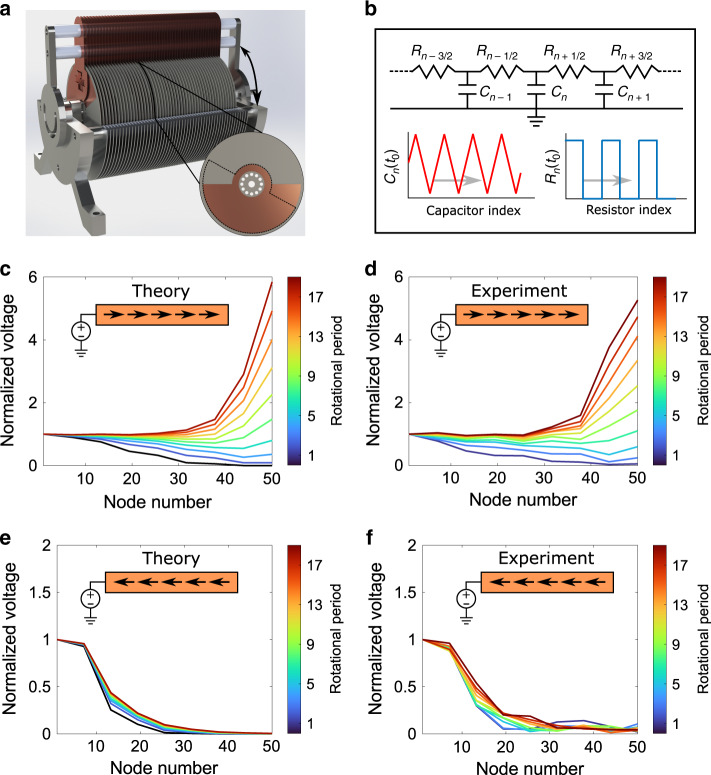
Theoretical and experimental demonstration of the temporal dynamics of the spatiotemporally varying diffusive medium. **a** Design of the experimental setup for the spatiotemporal metastructure with an inset showing the metallization of one of the 51 disks. The dotted lines show the relative position of the neighboring disk (rotated by angle $\pi /6$). The relative phase between the connections and the capacitors can be modified by rotating the top bar along the direction of the arrow. **b** Circuit representation of the chain of disks connected via angle-controlled switches and interdigitated by grounded stators to vary their capacitance in time. **c** Time evolution of the time-averaged voltage dictated by the analysis of the equivalent circuit for the realized metamaterial for $\phi = \pi /3$. **d** Experimentally measured time-averaged voltages along the sample for the same configuration as **c**. **e** Similar to **c** but **f**or $\phi = - \pi /3$. **f** Experimentally measured time-averaged voltages along the sample for the same configuration as **e**.

To demonstrate experimentally a proof-of-concept of this phenomenon, we have constructed a 1D spatiotemporally varying metamaterial for the case of electric charge diffusion (see Fig. 2a). This structure consists of 50 capacitors connected via 49 switches. Each capacitor consists of a circular disk (rotor) made of a semicircularly metalized printed-circuit board and a grounded stator. These metal plates produce a linear variation in the rotor’s capacitance directly related to the overlap between the metalized regions and the stators. In every two adjacent disks, one disk is rotated with angle $\pi /6$ with respect to its neighboring disk, resulting in spatial variation of the capacitance along the axis. A set of electrical contacts has been placed between each pair of adjacent disks, which serves to connect them when the metal portion of both disks and the contact is overlapping, thus forming a spatial variation of conductivity along the medium’s axis. The temporal variation of capacitance and conductivity can be achieved by rotating the ensemble of rotors at a constant angular velocity $\omega$, using a mechanical motor. The angle at which the contacts occur relative to the position of angle of capacitance can be modified at will, thus producing an arbitrary phase difference in the spatiotemporal variation of conductivity and the capacitance. The electrical contacts and variable capacitors in this case can be likened to spatiotemporally varying conductivity and permittivity (or capacity), respectively. This time-evolving structure can be described mathematically using homogenized expressions for the conductivity and inverse of capacitance (denoted by $C$), in the limit of a large number of disks, as

4 $$\sigma \left( {x,t} \right) = \sigma {\mathrm{{\Pi}}}\left( {\beta x - \omega t} \right)$$

5 $$g\left( {x,t} \right) = 1/C\left( {x,t} \right) = 1/({\mathrm{c}}_1{\mathrm{\Lambda }}\left( {\beta x - \omega t + \phi } \right) + c_0)$$

where ${\mathrm{{\Pi}}}\left( \cdot \right)$ and ${\mathrm{\Lambda }}\left( \cdot \right)$ represent normalized square and triangular dimensionless functions respectively. The constant offset of the capacity modulation $c_0$ is introduced in the design to ensure that the inverse of capacitance remains finite at all times. It is noteworthy that these spatiotemporal variations are consistent with a positive phase velocity along the *x* direction, but in the following we shall prove that this fact will not play any role in the effective bias velocity of the metamaterial.

In our setup, the first disk on one end of the sample (we call this the “left end”) is connected to a voltage source that maintains a constant potential between the first rotor and the stators. The equations governing the temporal dynamics of the system comprised of a series of switches alternated with parallel capacitors can be derived analytically in terms of the Kirchhoff laws, with the spatiotemporally varying capacitances and switches following a traveling triangular function and a traveling square function, as summarized in Fig. 2b. It can be shown that in the limit of a large number of disks, the Kirchhoff laws can be homogenized spatially, obtaining a differential equation in the form of Eq. (1). Details of such homogenization can be found in the Supplementary Note 2.

Although the experimental setup is presented for the case of electric charge diffusion, an analogous device could be envisioned for the case of thermal energy diffusion. One way to achieve this would be to substitute the variable capacitors with closed gas reservoirs whose volumes can be externally controlled by means of a set of pistons. The reservoirs are connected by variable thermal resistors, whose value can be controlled by mechanically modifying the cross-section in contact with the neighboring gas reservoirs. In this case, the variable thermal capacitors would follow, in the limit of high modulation frequencies, a series of adiabatic compression and expansion cycles, which can be modeled using the specific heat ratio of the gas used.

It is worth noting that due to the time modulation of the capacitance at any given node along the sample, the voltage at that point becomes also time-modulated even when the charge distribution has reached the steady state. However, we restrict our attention to the time-homogenized voltage, i.e., time-averaged voltage $\tilde V\left( {x,t} \right)$, which is proportional to the time-average charge density $\tilde q\left( {x,t} \right)$ (as mentioned before, this time averaging is over the period of the modulation, which is much shorter time than the diffusion time.)

In the following, we investigate two elucidating cases of the degree of control over the charge diffusion in our experimental setup.

In the first case, the effective velocity is chosen such that the charges are drawn from the source and accumulated at the opposite end of the sample. To achieve this, the relative phase between the spatiotemporal modulation of the capacitances and the switches has been chosen close to $\phi = \pi /3$.

The theoretical prediction (see Supplementary Note 3) for the temporal evolution of such system is shown in Fig. 2c, which clearly shows an accumulation of time-averaged charge/voltage towards the right end of the sample, in contrast to the conventional stationary state, i.e., flat evenly distributed charges, found in the absence of the modulation of the material parameters. Here we find a rapid growth in the time-averaged voltage (and associated charge) at the right end, which, in as few as five rotational periods, becomes larger than the source voltage. The rapid increase in charge accumulation then leads to a factor of 6 in less than 20 rotational periods of the system.

The experimental results for this case are shown in Fig. 2d, demonstrating a clear link with the theoretical results in Fig. 2c. We have been able to experimentally reproduce the voltage enhancement, i.e., factor of 6 within the first 20 rotational periods, predicted theoretically. We note that in real systems, large voltage accumulations may lead to arcing between neighboring conductors. Moreover, in our experiment, larger than six voltage enhancement could not be reliably measured due to the limitation of our instruments and thus not reported here (see Supplementary Note 4 for more information). As mentioned earlier, in the absence of such nonlinearities (e.g., arcing), the system will reach the steady-state accumulation, in which the force due to the effective velocity balances that of the diffusion mechanism that tries to minimize the charge accumulation. The shape of the charge distribution will then be dictated by the ratio between the diffusion coefficient and effective velocity achieved.

Let us now focus on the case in which the effective velocity points towards the source, which is achieved by imposing a relative phase of $\phi = - \pi /3$. As shown by the theoretical results in Fig. 2e, the voltage introduced by the source is clearly constrained within the first few elements of the structure, corresponding to those which are, at a certain value of the angle of rotation, directly connected to the source due to overlapping. Beyond this region, the voltage decays in such a way that the right end of the sample is not even perturbed by the presence of the voltage source. We have experimentally verified this, as shown by Fig. 2f. This implies that one can achieve isolation and “evacuation” of charges, heat, etc. from a region of space, not being constrained by the conventional diffusion mechanism. As for the accumulation phenomenon, the rate of decay is determined by the ratio between the effective velocity and the diffusion coefficient of the homogenized metamaterial, which can be controlled through the modulation amplitudes, the relative phase, and the wavelength of the spatiotemporal modulations.

We should highlight the fact that the only difference between the sample in Fig. 2c–f is the change in relative phase between the modulations of capacitance and conductivity, while maintaining every other aspect of the modulations the same. Therefore, as predicted from our theory, the sign of the modulations’ wavevector (i.e., the direction of the modulations) does not play any role in determining the direction of the biasing velocity.

## Discussion

**Fig. 3:**
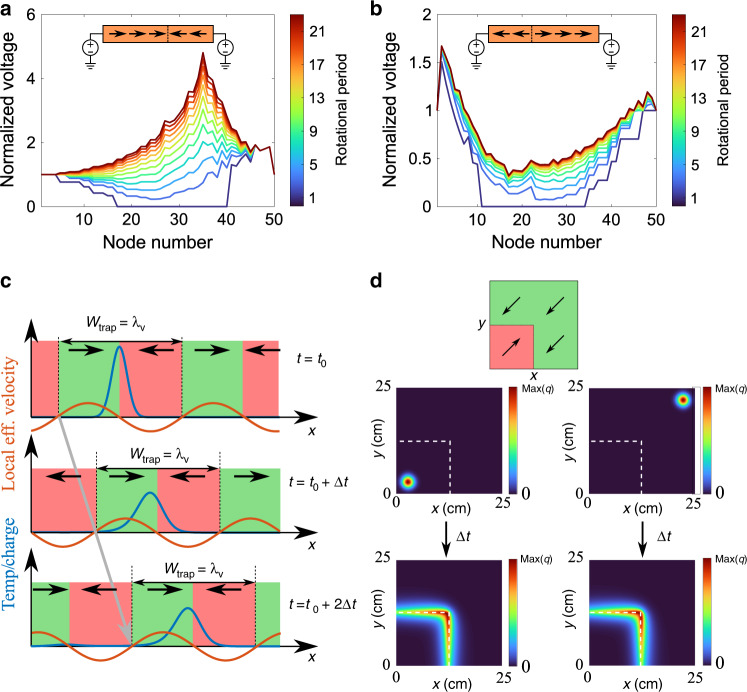
Accumulation of diffusive quantity at domain boundaries within the spatiotemporal metamaterial. Simulation results showing theoretical time evolution for the time-averaged voltage in a two-domain metamaterial with (**a**) converging (trap) and (**b**) diverging (anti-trap) effective velocities, achieved using different magnitudes and signs of the relative phase between modulations of the switches and the capacitances. In both cases, the steeper steady-state solution (right side in **a** and left side in **b**) is the result of a 1.5 factor in the amplitude of the capacitance modulation with respect to the opposite side. **c** Mobile diffusion trap based on the use of different frequencies and wavelengths for the spatiotemporal variations of conductivity and capacity, leading to propagating sinusoidal modulation of the effective biasing velocity capable of gathering and moving localized distributions of diffusive quantities. **d** Simulation results of the diffusive quantity for a corner-shaped trap based on a two-dimensional, two-domain metamaterial demonstrated under two different initial conditions.

We have demonstrated that the presence of interfaces between the metamaterial slab and insulators can be used to accumulate charge densities, leading to a voltage enhancement. For the second case, using numerical simulations, we now consider a different scenario, taking advantage of the flexibility and freedom in engineering the effective velocity. Specifically, we consider a discontinuous effective velocity, creating two domains within the metamaterial, across which the effective velocity changes sign. This is achieved by having two different phase differences between the spatiotemporal modulations of the conductivity and the capacitance. By doing this, one can achieve either accumulation or depletion of charges at the domain boundary. The simulation results for the time-evolving voltage distribution for a chain of 50 elements are shown in Fig. 3a for the case in which the effective velocity is positive in the left domain and negative in the right domain. Moreover, by choosing two different values of the magnitude of the effective velocity (through different modulation amplitudes of the capacitance), an asymmetric charge distribution is demonstrated numerically. Regardless of the asymmetry, our simulation shows a large and rapid accumulation of charge at the domain boundary. In contrast, as shown in Fig. 3b, when the effective medium velocities point away from the boundary, the charges are depleted from this region of interest. From Fig. 3a, b, it is clear that the position of the charge accumulation (i.e., diffusion trap) and depletion (i.e., diffusion anti-trap) can be engineered by the introduction of domain boundaries. Within our experiment, this could be achieved by splitting the sample such that the contacts of the two parts are independently positioned, creating a border across which the relative phase differences have opposite signs.

Although we have so far restricted ourselves to the case in which the biasing effective velocity is temporally invariant, it is possible to achieve temporally dependent effective velocity distributions. This would open the door to real-time reconfigurable diffusive systems that can take advantage of the accumulating and depleting capabilities already shown.

A solution to this scenario can come from the fact that the constant (in time and space) effective velocity is the result of the time homogenization (over the modulation period) of the product of the oscillating terms from the two spatiotemporal variations of capacity and conductivity. If one considers the case in which these two have different frequencies and wavelengths, one can show that this would lead to a homogenized diffusion equation in which the effective velocity also presents a spatiotemporal dependence consistent with the difference in wavevector and frequency (i.e., “beat frequency” and “beat wavelength”) between the spatiotemporal variations of the capacity and conductivity. With this, one can achieve sets of alternating points that accumulate/deplete any diffusive quantity (e.g., charge) that can also move in time and space along the structure (i.e., a moving set of interdigitated traps and anti-traps). As shown in Fig. 3c., a narrow Gaussian initial condition is introduced, such that the initial diffusive quantity distribution is limited within a trap. Also there, it is demonstrated that after a moving transient evolution the quantity of interest reaches a moving steady state, which is escorted by the moving diffusion trap when moving at small velocities compared to the scale of the velocity modulation. The fact that the effective velocity at every point of the metamaterial can be designed and modified in real time provides opportunities to achieve real time manipulation of diffusion phenomena in transient and steady-state scenarios.

Other exciting scenarios involve higher dimensions such as two- or three-dimensional metamaterials. One can imagine a planar spatiotemporal metastructure with the capability of accumulating diffusive quantities on a number of arbitrary points within the medium or at its boundaries. As a first proof of concept, we have simulated a two-dimensional, two-domain system in which a quarter of the plane presents a constant diagonal effective velocity, in the opposite direction to the other three quarters of the plane, as shown in Fig. 3d. Regardless of the initial distribution, we have shown that the diffusive quantity is trapped along a rather extraordinary shape of the corner. These results can be extended to introduce real-time modifications in the shape of the accumulating interface, leading to different steady-state distributions.

The approach presented here could be extended to systems in which the time-modulation is implemented via the propagation of waves, which interact with the medium modifying its properties as they advance. An example of this would be the propagation of pressure waves, which are already known to create thermoacoustic heat pumps when traveling through temperature gradients^32^.

In summary, we have investigated a mechanism involving metamaterials with spatiotemporally variations of conductivity and capacity, allowing for the biasing and control of the diffusion phenomenon. By combining domains of these metamaterials, we have demonstrated the ability to create attractors and barriers for the diffusive quantities both at external and internal boundaries. This approach may find applications in various branches of science and technology involving diffusive quantities such as charges, thermal energy, and particles, among others.

## Supplementary information

Supplementary Information

Description of Additional Supplementary Files

Supplementary Movie 1

Supplementary Movie 2

## Data Availability

All data are available in the main text and the Supplementary Information.

## Code availability

All codes produced during this research are available from the corresponding author at reasonable request.
