# Supplementary material for: Achieving asymmetry and trapping in diffusion with spatiotemporal metamaterials
Source: Nat Commun. 2020 Jul 24;11:3733. doi: 10.1038/s41467-020-17550-5 (PMC7381636; doi:10.1038/s41467-020-17550-5)
Supplement: Supplementary file 1 — Supplementary Information [file 41467_2020_17550_MOESM1_ESM.pdf]

Supplementary Information for

Achieving asymmetry and trapping in diffusion with spatiotemporal metamaterials

Camacho et al.

This PDF file includes:

Supplementary Notes 1 to 4

Supplementary References

Supplementary Figures 1 and 2

Supplementary Note 1: *Derivation of the homogenized advection-diffusion equation*

In the main text, the homogenization of the diffusive metamaterial is discussed when applied to the diffusion equation

$$\frac{\partial q(x,t)}{\partial t} = \frac{\partial}{\partial x}\left(\sigma(x,t)\frac{\partial}{\partial x}\big(g(x,t)q(x,t)\big)\right) \tag{S1}$$

where both the conductivity $\sigma(x,t)$ and the inverse of the capacitance $g(x,t)$ are functions of both space and time. Let us provide details of this mathematical procedure and the physical insight it provides. As a first step, we consider a sinusoidal modulation for these two quantities, as shown in the main text, such that $\sigma(x,t) = \sigma_0 + \sigma_1 \sin(kx - \omega t)$ and $g(x,t) = g_0 + g_1 \sin(kx - \omega t + \phi)$, with all parameters involved being constant. When one introduces these expressions into (1), one derives a differential equation shown as

$$\frac{\partial q(x,t)}{\partial t} = A(x,t)\frac{\partial^2}{\partial x^2}q(x,t) + B(x,t)\frac{\partial q(x,t)}{\partial x} \tag{S2}$$

where the coefficients $A(x,t)$ and $B(x,t)$ can be written as

$$\begin{aligned} A(x,t) = g_0\sigma_0 + \frac{1}{2}g_1\sigma_1\cos(\phi) + g_0\sigma_1\cos(kx-\omega t) + g_1\sigma_0\cos(kx-\omega t+\phi) \\ + \frac{1}{2}g_1\sigma_1\cos(2kx - 2\omega t + \phi) \end{aligned} \tag{S3}$$

$$\begin{aligned} B(x,t) = \frac{k}{2}(-g_1\sigma_1\sin(\phi) + 2g_0\sigma_1\sin(kx-\omega t) - 4g_1\sigma_0\sin(kx-\omega t+\phi) \\ + 3g_1\sigma_1\sin(2kx-2\omega t+\phi)) \end{aligned} \tag{S4}$$

As can be seen, $A(x,t)$ and $B(x,t)$ consist of a constant term in addition to the oscillatory terms. By restricting our analysis to the case in which $\sigma_1 \ll \sigma_0$ and $g_1 \ll g_0$, and homogenizing the quantities over the period of modulation, the characteristic time constant of the diffusive quantity $q(x,t)$ is then dictated by the constant factors of $A(x,t)$ and $B(x,t)$. This limited time response of the function $q(x,t)$ implies that the time-average of the oscillatory terms is null. This leads to a homogenized version of equation (3), which is presented in the main text.

Extensive simulations have been undertaken to corroborate our derived formula, and an example is presented here as Supplementary Figure 1 for the case of a Gaussian initial condition and in the Supplementary Figure 2 for a triangular case. For the same initial condition, one can reduce the frequency of the modulation until the temporal homogenization breaks down as shown for both cases. They show that, for these initial conditions, the homogenization effect is lost when the frequency is of the order of ten times the inverse of the diffusion time constant given by $\omega_{dif} = \sigma_0 g_0 \max\left|\frac{\partial^2 q\,(x,t)}{\partial x^2}\right|$ which in the case of Supplementary Figure 1 occurs at the initial condition with $\sigma_0 g_0 \max\left|\frac{\partial^2 q\,(x,t)}{\partial x^2}\right| \approx 0.01\ s^{-1}$.

Supplementary Note 2: *Derivation of the homogenized advection-diffusion equation from the circuit analysis*

By applying the Kirchhoff current law to the i-th node in Fig. 2B, one can derive

$$0 = \sum \mathrm{I} = i_{\mathrm{C,n}} + i_{R,n-\frac{1}{2}} - i_{R,n+\frac{1}{2}}$$

These currents can be written in terms of the time-dependent capacitances and conductivities ($S_n = 1/R_n$)

$$0 = \frac{\partial}{\partial \mathrm{t}}\big(C_n(t)V_n(t)\big) + S_{n-\frac{1}{2}}(t)\big(V_{n-1}(t) - V_n(t)\big) - S_{n+\frac{1}{2}}(t)(V_n(t) - V_{n+1}(t)) \qquad \text{(S5)}$$

One can easily recognize that the differences in voltage are just a discrete version of the derivative of the homogenized function given by $\frac{\partial V(x,t)}{\partial x}|_{x_{n-1/2}} = (V_n - V_{n-1})/\delta x$

$$0 = \frac{\partial}{\partial t}\big(C_n(t)V(x,t)\big) - \delta x S_{n-\frac{1}{2}}(t)\frac{\partial V(x,t)}{\partial x}|_{x_{n-\frac{1}{2}}} + \delta x S_{n+\frac{1}{2}}(t)\frac{\partial V(x,t)}{\partial x}|_{x_{n+\frac{1}{2}}} \qquad \text{(S6)}$$

where the first derivatives of $V(x,t)$ are evaluated at the position of the resistors. This expression again corresponds to a discretized derivative, such that it can be homogenized as

$$0 = \frac{\partial}{\partial t}\big(C_n(t)V(x,t)\big) - (\delta x)^2 \frac{\partial}{\partial x}\left(S(x,t)\frac{\partial V(x,t)}{\partial x}\right) \qquad \text{(S7)}$$

One can then express this in terms of the charges, as we did earlier when presenting the diffusion equation, in terms of the conductance and the inverse of the capacitance, obtaining

$$\frac{\partial}{\partial t}q(x,t) = (\delta x)^2 \frac{\partial}{\partial x}\left(S(x,t)\frac{\partial G(x,t)q(x,t)}{\partial x}\right) \qquad \text{(S8)}$$

Finally, (S8) can be homogenized by considering the conductivity and the inverse capacitance per unit length of the material such that

$$\frac{\partial}{\partial t}q(x,t) = \frac{\partial}{\partial x}\left(\sigma(x,t)\frac{\partial g(x,t)q(x,t)}{\partial x}\right) \qquad \text{(S9)}$$

which corresponds to the diffusion equation presented in the main text.

Supplementary Note 3: *Simulation methods*

Numerical codes have been implemented in MATLAB® to obtain the results in the main text based on the solution of both the exact (non-homogenized) and homogenized diffusion equations shown in Eqs. (1) and (3). These codes are based on discretization of both temporal and spatial domains. The solution for each time step is then found by calculating the time derivative of the unknown function based on its spatial distribution in the previous time step, an iterative procedure initialized with the given initial conditions (*1*). This technique is readily implemented in the Ordinary Differential Equation solvers in MATLAB® (*2*). The same time-discretization method has been applied for the replication of the experimental results by solving the Kirchhoff circuit equations as shown in Eq. (S5).

Supplementary Note 4: *Experiment: Voltage measurement*

The experimental setup described in the main text consists of 50 electrically isolated partially metalized disks interdigitated into a set of grounded stators. As such, each disk represents a capacitor whose capacitance varies according to its rotation angle. Connecting these metalized disks is a series of switches. Each switch consists of a spring leaf contact, which connects the two adjacent metalized disks when all three are properly aligned. This setup was excited through a voltage divider connected to an in-house customized 200-V constant voltage generator. Due to the low capacitance of the disks, the voltages were measured using a high impedance electrometer (PASCO ES-9078A), which was connected to a Feather M0 Adalogger board. The rotation of the disks was measured using an infrared (IR) reflectivity sensor, also connected to the board. The board was equipped with an SD card which saved the measured time-varying voltages from both the disk and the IR sensor. Prior to each experiment, the entire system was grounded to remove any residual static charge. Additionally, a thin coating of grease was applied to reduce friction which causes the creation of static charges. Data was measured for every third disk along the chain. This measurement was repeated at least three times per disk to confirm the validity of the data and was also performed in absence of voltage source to remove/calibrate friction-related static charges. These voltages were time-averaged over a single period to remove the fluctuations due to the locally varying capacitance.

Supplementary References

1. J. C. Butcher, *Numerical Methods for Ordinary Differential Equations* (John Wiley & Sons, New York, 2016).
2. L. F. Shampine, M. W. Reichelt, The MATLAB ode suite. *SIAM J. Sci. Comput.* **18**, 1–22 (1997).

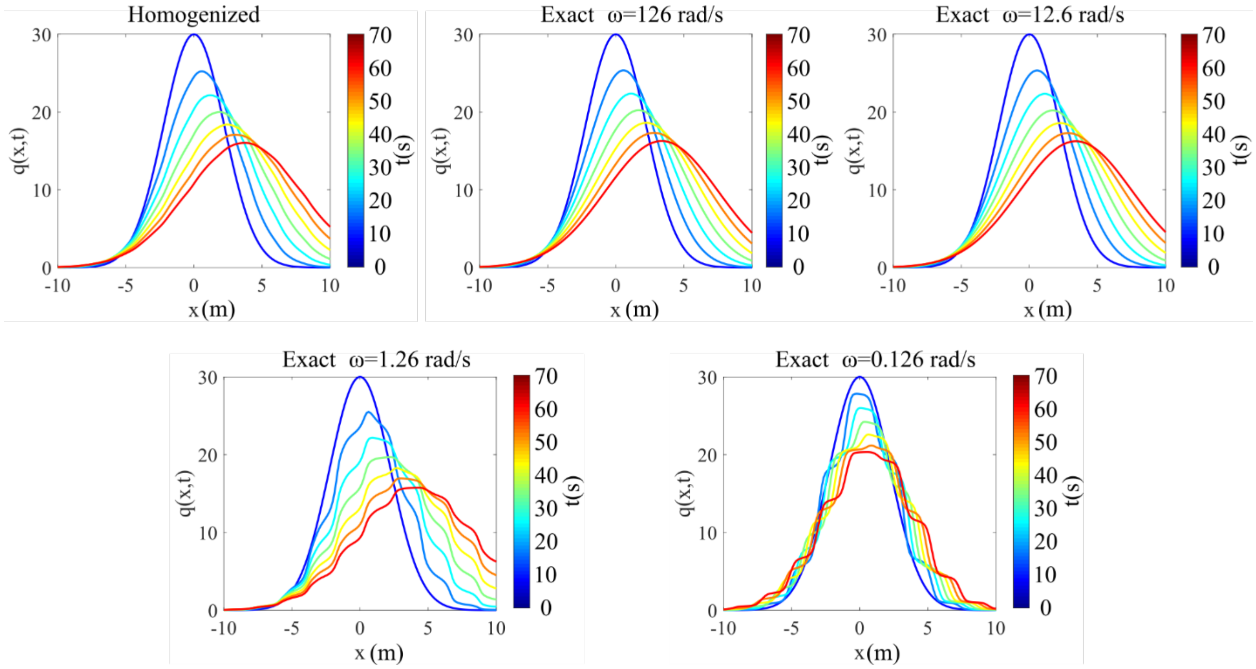

Supplementary Figure 1

Validation of the space-time homogenization procedure for the case of traveling-sinusoidal modulations of the conductivity and inverse of capacity the material for four values of the modulation frequency. The values of the other constants are $\sigma_0 = 0.1\frac{m}{\Omega}, \sigma_1 = 0.075\frac{m}{\Omega}, g_0 = 1\frac{m}{\mathrm{F}}, g_1 = 0.5\ \frac{m}{\mathrm{F}}, k = 3\frac{\mathrm{rad}}{\mathrm{m}}$. The curves shown correspond to snapshots of the function $q(x,t)$ every ten seconds with a Gaussian initial condition in the absence of source.

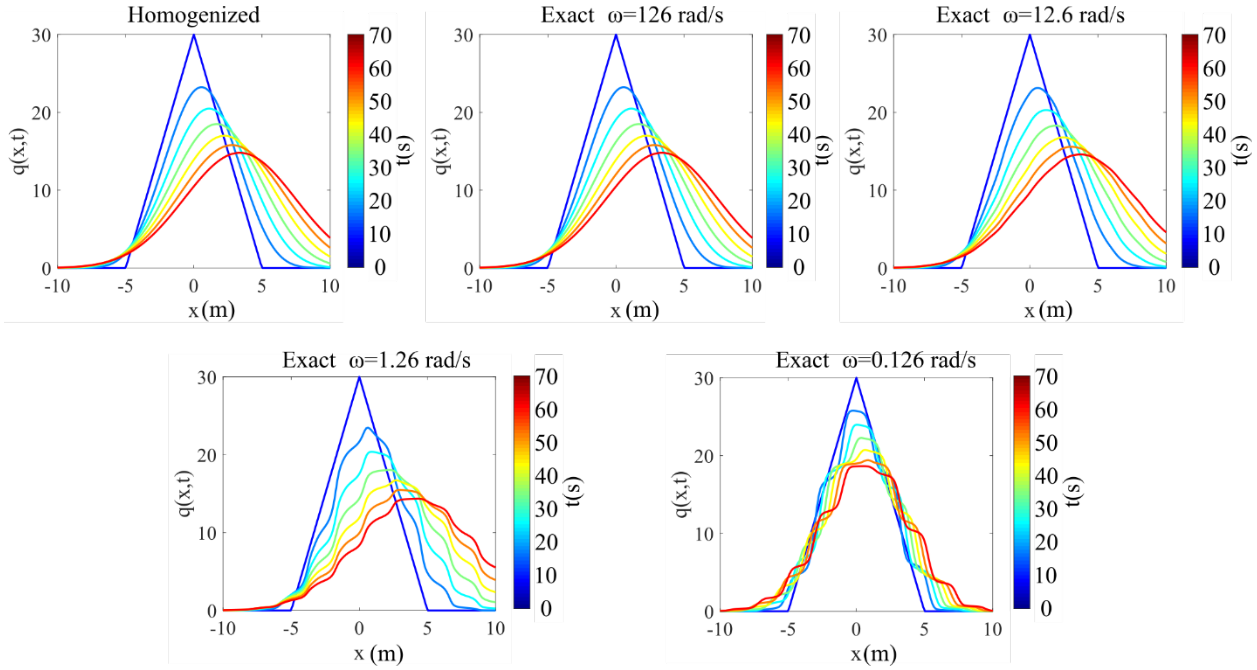

Supplementary Figure 2

Similar to Supplementary Figure 1, expect here we start with a triangular pulse initial condition.
