# Supplementary material for: Achieving asymmetry and trapping in diffusion with spatiotemporal metamaterials
Source: Nat Commun. 2020 Jul 24;11:3733. doi: 10.1038/s41467-020-17550-5 (PMC7381636; doi:10.1038/s41467-020-17550-5)
Supplement: Supplementary file 2 — Description of Additional Supplementary Files [file 41467_2020_17550_MOESM2_ESM.pdf]

Description of Additional Supplementary Files

File Name: Supplementary Movie 1
Description: Time evolution of the diffusion process in a spatiotemporally modulated metamaterial showing the independence of the sign of the effective velocity with respect to the propagation direction of the modulations. The value of the material parameters are $\sigma_0=0.2\ m/\Omega, \sigma_1=0.15\ m/\Omega, g_0=1\ m/F, g_1=0.5\ m/F, k=5\ rad/m, \omega=1.68\ rad/s, \varphi=-\pi/2$.

File Name: Supplementary Movie 2
Description: Time evolution of the diffusion process in a spatiotemporally modulated metamaterial showing the independence of the sign of the effective velocity with respect to the propagation direction of the modulations. The value of the material parameters are $\sigma_0=0.2\ m/\Omega, \sigma_1=0.15\ m/\Omega, g_0=1\ m/F, g_1=0.5\ m/F, k=5\ rad/m, \omega=1.68\ rad/s, \varphi=\pi/2$.
.
