# Supplementary figures and images for: Achieving asymmetry and trapping in diffusion with spatiotemporal metamaterials
Source: Nat Commun. 2020 Jul 24;11:3733. doi: 10.1038/s41467-020-17550-5 (PMC7381636; doi:10.1038/s41467-020-17550-5)

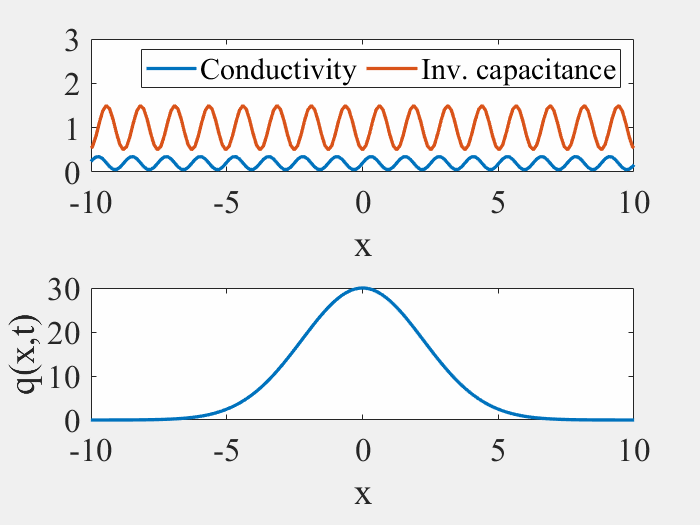

Supplement: Supplementary file 3 — Supplementary Movie 1 [file 41467_2020_17550_MOESM3_ESM.gif]

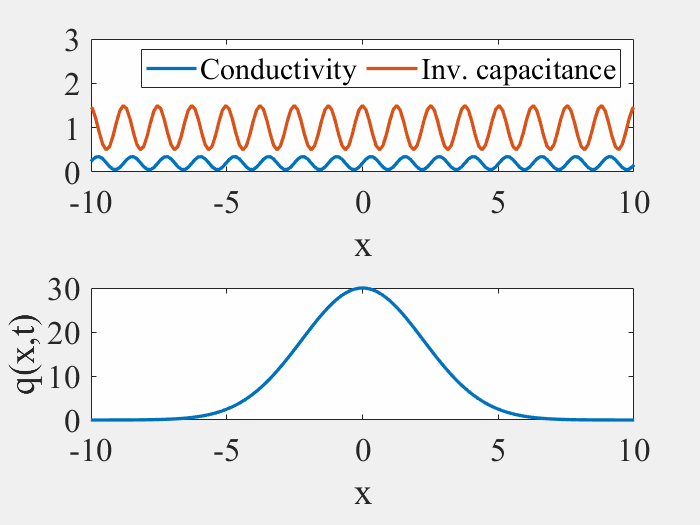

Supplement: Supplementary file 4 — Supplementary Movie 2 [file 41467_2020_17550_MOESM4_ESM.gif]
